# Uncovering the Underworld of Axial Spondyloarthritis

**DOI:** 10.3390/ijms24076463

**Published:** 2023-03-30

**Authors:** Sergio Del Vescovo, Vincenzo Venerito, Claudia Iannone, Giuseppe Lopalco

**Affiliations:** 1 Department of Precision and Regenerative Medicine and Ionian Area (DiMePRe-J), Polyclinic Hospital, University of Bari, 70124 Bari, Italy; 2 Division of Clinical Rheumatology, ASST Gaetano Pini-CTO Institute, 20122 Milan, Italy

**Keywords:** axial spondyloarthritis, innate immunity, cytokines, biological agents, personalized medicine

## Abstract

Axial spondyloarthritis (axial-SpA) is a multifactorial disease characterized by inflammation in sacroiliac joints and spine, bone reabsorption, and aberrant bone deposition, which may lead to ankylosis. Disease pathogenesis depends on genetic, immunological, mechanical, and bioenvironmental factors. HLA-B27 represents the most important genetic factor, although the disease may also develop in its absence. This MHC class I molecule has been deeply studied from a molecular point of view. Different theories, including the arthritogenic peptide, the unfolded protein response, and HLA-B27 homodimers formation, have been proposed to explain its role. From an immunological point of view, a complex interplay between the innate and adaptive immune system is involved in disease onset. Unlike other systemic autoimmune diseases, the innate immune system in axial-SpA has a crucial role marked by abnormal activity of innate immune cells, including γδ T cells, type 3 innate lymphoid cells, neutrophils, and mucosal-associated invariant T cells, at tissue-specific sites prone to the disease. On the other hand, a T cell adaptive response would seem involved in axial-SpA pathogenesis as emphasized by several studies focusing on TCR low clonal heterogeneity and clonal expansions as well as an interindividual sharing of CD4/8 T cell receptors. As a result of this immune dysregulation, several proinflammatory molecules are produced following the activation of tangled intracellular pathways involved in pathomechanisms of axial-SpA. This review aims to expand the current understanding of axial-SpA pathogenesis, pointing out novel molecular mechanisms leading to disease development and to further investigate potential therapeutic targets.

## 1. Introduction

**Figure 1:**
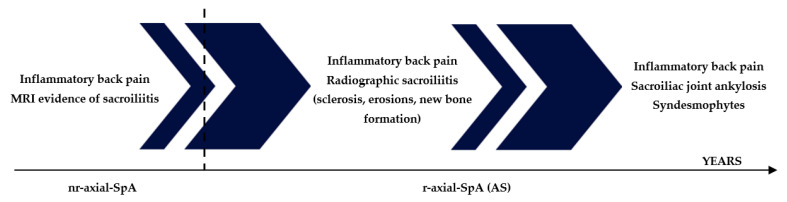
Axial-SpA may evolve over time, from an early phase without radiologically detectable changes in the pelvic X-ray (nr-axial-SpA) toward a late phase in which structural changes such as sclerosis, erosions, and new bone formation in the sacroiliac joints and in the spine occur (r-axial-SpA).

Axial spondyloarthritis (axial-SpA) is a multifactorial disease arising from several factors whose pathogenesis is not yet completely clarified and is characterized by inflammation in sacroiliac joint and spine, bone reabsorption, and aberrant bone deposition, which may lead to ankylosis. Axial-SpA is the modern term used to identify a particular form of SpA with a predominant axial involvement, including two distinct clinical pictures, nonradiographic axial SpA (nr-axial-SpA) and radiographic axial-SpA (r-axial-SpA), traditionally termed ankylosing spondylitis (AS), depending on whether the axial disease involvement has determined visible radiographical lesions on X-ray [1]. These two clinical entities may be considered as a disease continuum marked by an early phase, named nr-axial-SpA, in which clinical manifestations occur without radiographical evidence [2]. Patients typically present with inflammatory back pain (IBP) starting before 45 years of age. Pain usually lasts more than 3 months, worsens at night and with rest, whereas it improves with exercise [3]. At this stage, the disease may cause inflammatory abnormalities of sacroiliac joints detectable on magnetic resonance imaging (MRI) such as bone marrow oedema (BME). Of note, nr-axial-SpA patients may develop, during their life, structural changes that could be disclosed on pelvic X-ray, configuring the clinical framework of r-axial-SpA. On the contrary, r-axial-SpA is characterized by X-ray-detectable sacroiliitis and spinal involvement. Later stages of the disease may be marked by complete ankylosis of the sacroiliac joints as well as the spine due to syndesmophyte formation [2]. The clinical disease course including the early and late stages of axial-SpA is depicted in Figure 1. Several components including genetic, environmental, biomechanical, and immunological factors are involved in disease pathogenesis [4]. A pivotal role may be attributable to the innate immune system, in contrast with other systemic autoimmune diseases, such as rheumatoid arthritis, where adaptive immunity, alongside the innate counterpart, holds a key role [5,6]. Therefore, abnormal activity of innate immune cells has been demonstrated, including γδ T cells, type 3 innate lymphoid cells, macrophages, and mucosal-associated invariant T cells, at tissue-specific sites prone to the disease [7]; this may also be the bridge explaining the strong relationship between the gut and joint inflammation (gut–joint axis). In fact, these patients may experience gut disorders as demonstrated by the high prevalence of subclinical bowel inflammation [8]. Currently, several therapies have been approved for axial-SpA, especially TNF-α inhibitors, IL-17 inhibitors, and JAKs inhibitors, targeting critical molecules involved in disease pathogenesis [9]. However, there is an actual need for new therapeutic approaches, as patients with axial-SpA may not respond or be intolerant to treatment. The aim of this review is to consider the most important genetic, cellular, and molecular factors that perform a key role in developing the disease phenotype, in particular from an immunological point of view. Even if disease pathogenesis has not been fully elucidated yet, current knowledge of its pathogenetic mechanism has advanced in recent years. On this basis, several effective therapeutic instruments are now available in axial-SpA treatment.

## 2. HLA-B27: The Main Genetic Player in Axial-SpA Pathogenesis

One of the most important factors involved in axial-SpA development is surely represented by several genetic elements, among which HLA-B27, the major histocompatibility complex (MHC) class I allele, represents the one with the most important weight [10]. This particular allele is shared by 85–90% of axial-SpA patients, even though only 5% of people carrying HLA-B27 in their genetic background will develop axial-SpA during their life [11]. This allele is profoundly polymorphic: 392 alleles (https://www.ebi.ac.uk/ipd/imgt/hla/alleles/ (accessed on 1 November 2022)) have been described over time, each with minimum differences with the others, mainly of one or a few amino acids in the whole protein sequence. The molecular features of HLA-B27 and its subtypes seem to have importance in relation to the theories linking this molecule to axial-SpA. Indeed, only specific HLA-B27 subtypes seem to be involved in the genetic predisposition to AS: in particular, HLA-B27*02, HLA-B27*04, and HLA-B27*05 [12].

On the other hand, other polymorphic forms of this MHC class I molecule such as HLA-B27*06 and HLA-B27*09 [12] are unrelated to the disease and differ from the disease-related subtypes only in one or more residues of the F pocket in the peptide-binding groove. Guiliano B. et al. studied HLA-B27*05 and mutants resembling HLA-B27*06 and 09 subtypes. HLA-B*27:09 has only one change from aspartic acid to histidine at p116, while the 06 subtype expresses a change from histidine to aspartic acid and from aspartic acid to tyrosine at p114 and p116, respectively, when compared to HLA-B27*05. Both p114 and p116 take part in determining the F pocket structure, which is important for binding the carboxy-terminal motif of MHC class I-associated peptides. Not only does this region have an important role in the peptide binding, but their observation showed that the F pocket residues might affect dimerization and chaperone association and alter the maturation rates of HLA-B27 subtypes. All these factors have been linked to the development of disease pathogenesis [13]. Moreover, the molecular stability of HLA-B27*05 and *09 subtypes are different. Hence, the *05 subtype is more stable versus thermal denaturation than the other, perhaps making the *05 subtypes prone to cellular accumulation, in contrast with the *09 subtype which is less stable and could be easily degraded. In addition, the amino acid C67 is much more exposed in the *05, which could potentially explain how it more easily forms C67-mediated disulphide bonded dimers [14,15], albeit other authors found no differences in the ability to form homodimers between disease-associated and -unassociated subtypes [16]. The minimal differences between these subtypes seem to modulate HLA-B27 conformational flexibility. Indeed, the disease-associated subtypes *04 and *05 have been shown to have increased plasticity, rather than the 06 and *09 subtypes which are less flexible. It has been suggested that molecular plasticity inversely correlates with the efficiency of negative T cell selection within the thymic medulla. This process may be impaired when the T cells confront a highly flexible binding groove, causing the failure of negative selection against self-peptide [17,18]. The surviving CD8^+^ T lymphocytes would then recognize these peptides in peripheral tissues, in contrast to what happens in the thymus, due to increased peptide concentration in key peripheral tissues or post-translationally modifications that take place in specific tissues [19].

Over time, many efforts were made to understand the mechanism by which this MHC I molecule could interfere with axial-SpA pathogenesis. Therefore, three different theories have been proposed to explain HLA-B27 role: (1) the arthritogenic peptide, (2) HLA misfolding and accumulation, (3) HLA-B27 homodimers on cell surface. The gene HLA-B27 encodes, as said, for an MHC class I molecule, which is a complex of a heavy chain with three α-domains (α1, α2, α3) and a β_2_-microglobulin chain. Its peptide-binding groove can bind short peptides that are exposed at the cellular surface after appropriate proteolytic degradation. Therefore, this trimolecular complex (MHC heavy chain, β_2_-microglobulin, short peptide) can interact with the T cell receptor (TCR) of CD8^+^ T lymphocytes [20].

Regarding the arthritogenic peptide theory, HLA-B27 could present to CD8^+^ T lymphocytes certain short peptides (“arthritogenic peptide”) from bacterial sources, similar to self-peptide expressed by disease-targeted tissues (i.e., spine and peripheral joints), triggering an immune cross-reaction. An adaptive immune reaction driven by these lymphocytes could then follow and explain how tissue damage originates. The validity of this theory has been hard tested and seriously put in doubt. In fact, in HLA-B27^+^ transgenic rats lacking CD8^+^ cells, disease developed, suggesting that this cellular line is not essential for the pathogenesis. Moreover, it is well-known that HLA-B27^-^ people could be equally affected by axial-SpA. However, several data suggest that this theory should not be ruled out. Firstly, high levels of CD8^+^ T cells were demonstrated in the enthesis of SpA patients [21]. Moreover, CD8^+^ T cells were seen to be increased in the synovial fluid (SF), with a cytotoxic phenotype [22]. The responsiveness of HLA-B27-restricted CD8^+^ T cells against self-peptide was also demonstrated in AS, particularly towards cartilage-derived self-peptide [23]. It has also been reported that CD8^+^ T cell function could be affected by AS-associated T-box transcription factor *TBX21* variants which alter T-bet expression [24]. In addition, Faham et al., through an NGS-based approach, discovered several TCRβ motifs enriched in B27^+^ AS patients. These motifs are present at a higher frequency in AS patients when compared to healthy individuals carrying HLA-B27. Therefore, these findings suggest the existence of specific antigens involved in axial-SpA pathogenesis [25]. In another recent study, 37 HLA-B27^+^ AS patients shared 10 closely related CD8^+^ TCR clones, while these clones were detected only in 4 out of 19 HLA-B27^+^ controls, putting in evidence for their possible involvement in disease onset [26].

Another possible explanation of the HLA-B27 role is the HLA misfolding and accumulation in the Endoplasmic Reticulum (ER) due to its chemical properties. Usually, once synthesized, this molecule forms a heterotrimeric structure, exposed on the cell surface following intracellular trafficking. Differences in the F pocket may dramatically affect the maturation rates of HLA-B27 subtypes. It has been demonstrated that HLA-B27*06 and *09 may mature at maximum within 90 min, while *05 has slower maturation rates, as it needs about 3.5 h. This exposes its cysteine residues (in particular Cys67) to oxidation changes in the ER for a longer time, leading to the development of disulphide bonds and the accumulation of HLA-B27*05 in the ER [13]. ER accumulation of misfolded HLA-B27 has several consequences. Firstly, it activates ER-associated protein degradation (ERAD) pathways which lead to the removal of a part of misfolded proteins to prevent ER stress [27,28]. Indeed, excessive accumulation of misfolded proteins may trigger the unfolded protein response (UPR), which in turn leads to the suppression of protein translation, upregulation of ER chaperones, activation of transcription factors such as CCAAT-enhancer-binding protein homologous protein (*CHOP*), and activation of proinflammatory pathways [29]. In particular, CHOP can enhance the IL-23/IL-17 pathway by increasing the expression of the *p19* unique subunit mRNA of IL-23 [30]. However, contrasting evidence has been collected in regard to UPR involvement in axial-SpA pathogenesis. On the one hand, UPR is activated in macrophages derived from the bone marrow of HLA-B27 transgenic rats with inflammatory disease [31], hence, upregulating the IL-23/IL-17 pathway [32,33]. On the other hand, macrophages from blood and synovial tissue of HLA-B27^+^ SpA patients did not show elevated ER stress markers [34]. Moreover, in the gut of AS patients, UPR was found to be not upregulated, whereas IL-23 expression in lamina propria mononuclear cells was derived from autophagy upregulation [35]. In this regard, autophagy seems to have a prominent role, along with ERAD, in removing misfolded and accumulated HLA-B27 aberrant forms. In fact, in bone marrow-derived macrophages from HLA-B27 transgenic rats, activation of autophagy with rapamycin eliminated a great part of misfolded HLA-B27. The same study also suggested that impaired ubiquitination of HLA-B27 may play a role in the accumulation of misfolded disulphide-linked dimers [36]. Another independent study provided important information about autophagy involvement in axial-SpA: mRNA of autophagy-related genes such as *ATG5* and *ATG12* were downregulated in AS patients and their expression was inversely correlated with disease activity. Nevertheless, the lncRNA *GAS5*, which positively correlated with *ATG5*, *ATG12*, and other autophagy-related genes, turned out to be downregulated. Moreover, autophagy seems to not be involved in B27*09 clearance, in contrast with the disease-associated B27*04 [37]. These findings suggest a possible therapeutic implication of acting on the modulation of autophagy pathways through therapeutic agents such as the mTOR inhibitor rapamycin.

Another possible explanation for HLA-B27 involvement in the disease pathogenesis is the formation of HLA-B27 homodimers (B27_2_), which can occur during the endosomal-dependent HLA-B27 recycling pathway [38]. Surface B27_2_ could then modulate the immune response through interaction with several receptors such as leucocyte immunoglobulin (Ig)-like receptors LILR and killer cell immunoglobulin (Ig)-like receptors KIR [39,40]. In particular, the interaction between B27_2_ and the receptor KIR_3_DL_2_, on the surface of NK cells and IL-17 producing CD4^+^ T cells seems to play a critical role. In fact, KIR_3_DL_2_ could be engaged by these homodimers more strongly than other classic MHC class I molecules [41]. On the one hand, B27_2_ may engage the receptor on NK cells surface and enhance their proliferation and cytotoxicity, activating the PI3K/Akt pathway [42,43]. On the other hand, B27_2_ can bind the receptor on the surface of T_H_17 cells, enhancing their survival and proliferation and stimulating IL-17 release, a key cytokine involved in axial-SpA pathogenesis [44].

In recent years, some evidence also suggests a role for HLA-B27 in bone formation, a distinctive feature of AS. Some authors proposed an indirect role for this molecule through its influence on the inflammatory state [45]. Other studies found that the presence of this haplotype, and not specifically the disease, correlates with lower sclerostin- and Dickkopf 1 (Dkk-1)-serum levels. These two proteins are antagonists of the Wnt-catenin pathway, fundamental for new bone formation. Moreover, a relationship between HLA-B27 and higher levels of Indian hedgehog (IHH), a protein involved in endochondral ossification, have been demonstrated as well [46]. B27 may also interfere with BMP/TGFβ pathways. It can reduce the ALK2 function, which in turn is able to antagonize the action of proteins such as BMP or TGFβ, which are involved in the process of new bone formation [47]. Of note, HLA-B27 misfolding mediates the activation of the inositol-requiring 1 (p-IRE1)/spliced X-box-binding protein 1 (sHBP1) pathway, capable of upregulating the retinoic acid receptorβ (RARβ)/tissue-nonspecific alkaline phosphatase (TNAP) axis in AS mesenchymal stem cells. This pathway, together with the action of TNAP, is essential for spinal ankylosis and syndesmophyte formation [48].

**Figure 2:**
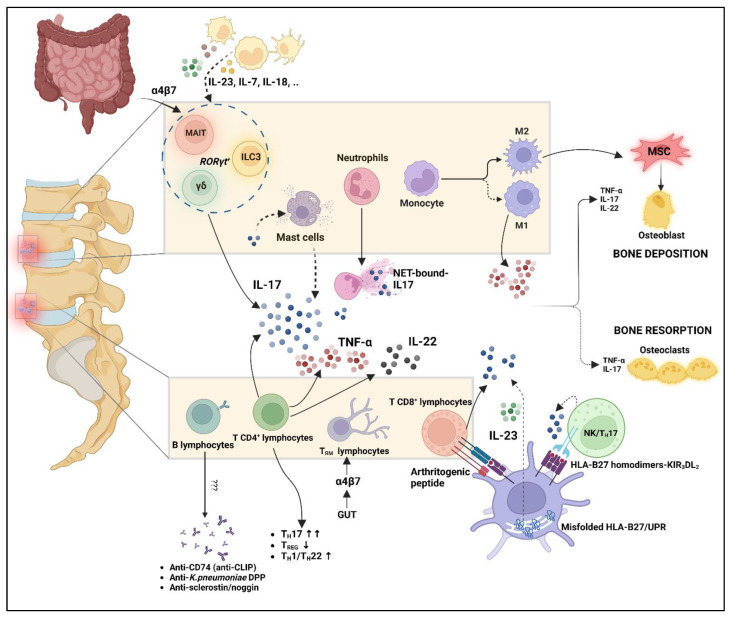
Interplay of innate and adaptive immune system in axial-SpA pathogenesis. Mast cells are not able to produce IL-17; however, they may capture it from extracellular environments and release it after unknown stimulus. NET-bound-IL17 has been demonstrated useful, along with its capability to induce osteogenic differentiation of MSC. Myeloid cells can produce a large amount of proinflammatory cytokines, which may also act on innate-like lymphocytes and innate lymphoid cells such as MAIT, *ILC3*, and γδ T cells. Not only these cells, but also the T_RM_ lymphocytes, may concentrate in axial enthesis following migration from the gut, as suggested by intestinal homing markers such as the α4β7 integrin on their surface (gut–joint axis). T cells play a predominant role among adaptive immunity, with an imbalance between T_H_17 and T_REG_ cells, but also T_H_1/22 upregulation. T CD8^+^ cells may be activated after presentation of arthritogenic peptide by HLA-B27. HLA-B27 may also contribute to IL-23/IL17 upregulation due to UPR activation and B27_2_ binding of KIR_3_DL_2_ receptors on the NK/ T_H_17 cell surface. This cellular interplay leads to an aberrant production of proinflammatory cytokines such as TNF-α, IL-17, and IL-22, which in turn may dysregulate bone homeostasis along with M2 macrophages. B lymphocytes are traditionally considered less relevant even if antibody production has been demonstrated. MAIT: mucosal-associated invariant T cells; *ILC3*: innate lymphoid cells 3; MSC: mesenchymal stem cells; UPR: unfolded protein response. “Created with BioRender.com”.

Another important property of HLA-B27 in axial-SpA is its epistatic interactions with the aminopeptidase ERAP1. Indeed, polymorphisms in the two aminopeptidases ERAP1 and ERAP2 may account for about 15–20% of genetic predisposition in axial-SpA [49]. ERAP1 and ERAP2 trim the peptides in the ER to shorter peptides, making them able to be presented by MHC-I molecules [50]. ERAP1 also has another action, it cleaves cytokine receptors on the cell surface, such as TNFR1, IL6R2, IL1R2, thus, modulating the immune function, even if this function seems to be not involved in disease pathogenesis [51]. However, the ERAP1 role in AS predisposition has been seen only in HLA-B27^+^/B40^+^ patients [52]. This underlines the importance of a correct peptide trimming and a correct recognition and peptide presentation by the MHC-I, reinforcing the arthritogenic peptide hypothesis. The most common ERAP1 AS-associated SNPs (rs2287987, rs30187, rs10050860, rs17482078, and rs27044) [53] determine a change in the HLA-B27 peptidome [54]; therefore, an impaired ERAP enzymatical activity leads to suboptimal peptides that should be bound by the B27 inclining to disease [55]. On the contrary, contrasting evidence has been collected about the possible correlation between ERAP SNPs and their influences on HLA-B27 misfolding, UPR, and B27_2_ accumulation on the cell surface [56,57,58,59]. The pathogenic role of HLA-B27 in axial-SpA is depicted in Figure 2.

## 3. Role of Innate Immunity

The role of several cellular types belonging to innate immunity in axial-SpA pathogenesis has been widely demonstrated.

Firstly, mast cell infiltration has been proven in tissues affected by this disorder [60]. Mast cells are a prominent source of secreted IL-17A; albeit they are not able to synthesize this cytokine, they may capture IL-17A from the extracellular environment through receptor-mediated endocytosis and store it in intracellular granules. Accordingly, IL-17A is rapidly available in its active form in these cells and could be secreted following a proper stimulus. Currently, it is unknown what triggers mast cells to secrete the stored IL-17A. IL-17F also, in smaller concentrations than IL-17A, has been found stored in these intracellular vesicles [61]. Mast cells may be targeted by some c-Kit inhibitors such as nilotinib and imatinib, which could induce mast cell apoptosis. On this basis, a small clinical trial was conducted on SpA patients with nilotinib showing some positive effects only in patients affected by peripheral-SpA, with no efficacy evidenced in axial-SpA [62].

Neutrophils seems to be also involved in axial-SpA pathogenesis, as their amount is significantly higher in patients vs. healthy donors. Moreover, a correlation between neutrophils and the Bath Ankylosing Spondylitis Disease Activity Index (BASDAI) has been demonstrated. Patients with higher neutrophils have high BASDAI scores, whereas lower neutrophil levels are associated with lower BASDAI scores [63]. The neutrophils ability to produce IL-17A/F is still controversial, as contrasting evidence has been collected nowadays [64]. A study found that an important percentage of IL-17 positive cells in facet joints from axial-SpA was represented by neutrophils [65]. Moreover, *IL-17* mRNA along with the *RORγT* mRNA, the latter being critical for IL-17 production, have been demonstrated to be produced in AS patient neutrophils. Of note, both of these two mRNAs were expressed in AS neutrophils at higher levels than healthy controls [66]. Recently, the neutrophil extracellular traps (NETs)- role in AS has been evidenced by independent studies [66,67,68]. NETs are extracellular structures made up of several proteins assembled on a decondensed chromatinic scaffold whose role is to neutralize and kill several microbes. NETs dysregulation has been linked to the pathogenesis of several immune-related diseases, such as systemic lupus erythematosus (SLE) [69]. Papagoras et al. first demonstrated that NETs production is enhanced in AS subjects. Furthermore, they found NETs were associated and bound with proinflammatory cytokines such as IL-17A and IL-1β. In addition, NET-bound IL-17A could promote mesenchymal stem cell (MSCs) differentiation toward an osteogenic phenotype, thus increasing osteogenesis, a hallmark of axial-SpA. However, in the same study, free serum IL-17A was not able to induce this differentiation, which may probably occur because of the high localized concentrations of this cytokine due to NETs structure action, which remarkably increases the bioactivity of IL-17A in AS. IL-17A neutrophil production was promoted mainly by IL-1β and NET-bound IL-1β, whereas no stimulatory role was found for TNF-α and IL-23. For this reason, IL-1β inhibition with the interleukin-1 receptor antagonist anakinra would reduce the osteogenic differentiation of MSCs via downregulation of IL-17 bound to NETs [66]. Ruiz-Limon et al. also demonstrated spontaneous NET formation, proposing a role for some circulating cell-free NETosis-derived products as biomarkers that could discriminate between axial-SpA patients and healthy donors and are also related to clinical inflammatory parameters [68].

Moreover, macrophages were found to be one the most abundant cell population types in inflammatory infiltrates in the sacroiliac joint of axial-SpA patients [70]. In AS, macrophages are more prone to produce inflammatory cytokines such as TNF-α, and express greater concentrations of IL-1β as seen when stimulated with proinflammatory stimuli such as BzATP [71]. Entheseal and gut myeloid cells both have a main role in the production of IL-23. In this regard, enthesis hosts a particular CD14^+^ myeloid cellular population that may produce this cytokine and other ones such as TNF-α or IL-1β, both in the enthesis soft tissue and perientheseal bone [72]. Furthermore, in the gut a distinct population of CX_3_CR1^+^CD45^+^ cells is able to produce IL-23, thus stimulating innate lymphoid cells 3 (ILCs3), which are in turn involved in axial-SpA pathogenesis. This CX_3_CR1^+^CD45^+^ population, with a proinflammatory transcriptomic profile, is expanded in AS gut but also in peripheral blood (PB), SF, and BM, expressing the CCR9, a marker of intestinal homing. Therefore, these cells, activated in the gut, may migrate through the blood to disease-affected tissues [73]. Nevertheless, both in the microenvironment of the diseased local tissue and in the PB of advanced AS patients, a predominant rate of CD163^+^ M2 macrophages was found [74]. This could confirm the general thought that advanced phases of the disease are characterized by the predominance of abnormalities such as tissue repair and remodelling. The novel mechanism by which M2 macrophages could influence bone formation in AS has just been unveiled. Indeed, M2 cells can release extracellular vesicles (EV) rich in *miR-22-3p* which in turn could regulate osteogenic pathways in MSCs. This miRNA seems to stimulate the Wnt/β-catenin pathway through inhibition of period circadian protein 2 (PER2) [75]. The pathway, furthermore, could be activated by the macrophage migration-inhibiting factor 2 (MIF2), which is fairly increased in serum of AS patients. This M2-polarized cytokine can increase macrophage TNF-α production and osteogenesis through β-catenin activation [76]. MIF seems to have great importance in AS pathogenesis, and its inhibition could be a novel therapeutic target in order to control bone formation [77].

*Innate lymphoid cells (ILC)* and *Innate-like lymphocytes (ILL)* have been recognized as important cellular actors in several chronic inflammatory diseases, including axial-SpA.

Indeed, *ILC3* seem to be involved in AS pathogenesis from several pieces of evidence. These cells are located mainly at mucosal barriers, in particular in the gut where they regulate interactions with the microbiome and gut homeostasis [78,79]. Two distinct *ILC3* populations including NKp44^+^ and NKp44^-^ cells may be recognised. The former produces mainly IL-22, while the latter produces mainly IL-17, thus resembling Th22 and Th17 cell functions [80]. Since they are not able to recognize pathogens directly in the gut, they could be activated indirectly, through interactions with macrophages, dendritic cells, and several proinflammatory cytokines [81]. However, although they are traditionally regarded as mainly statically located at the gut barrier, their presence was demonstrated also in several peripheral tissues such as the enthesis of healthy donors [82]. Moreover, in AS patients, this cell population was found to be expanded in PB, SF, and BM, its increase being correlated with BASDAI. Although it is unclear where these cells come from, some evidence suggests that they could migrate from the gut to these tissues. In fact, it has been seen that these cell in PB, SF, and BM express high concentrations of the intestinal homing molecule α4β7. In addition, its specific counter-receptor, the mucosal vascular addressin cell adhesion molecule 1 (MAdCAM1) was upregulated in the gut and in the BM of AS patients [83].

Among *ILLs*, mucosal-associated invariant T cells (MAIT) are acquiring increasing relevance over the last few years [84]. These cells are found in abundance at mucosal and epithelial barriers, in particular the gut lamina propria, but also in the liver, where they represent among 20–45% of resident T cells [85]. The importance of these cells in axial-SpA pathogenesis and their gastrointestinal predominant origin further underlines the tight connection existing between joint and gut inflammation often found in these patients [8]. MAIT could be activated through two different pathways: (1) recognition of the monomorphic MHC class-1 related protein MR1 bound to nonpeptide antigens by the semi-invariant MAIT TCR or (2) cytokine stimulation upon receptor expressed on their cellular surface such as IL-7R, IL-23R, IL-12R, and IL18R [86]. MAIT concentrations in PB from AS patients are decreased, at the same time being increased in inflamed tissues such as SF of inflamed joints. Here, they show an activated phenotype that seems to be dependent more on cytokine stimulations rather than TCR stimulation [87]. These cells can produce T_H_1 and T_H_17 type cytokines including TNF-α and IL-17. Although in AS samples no difference was seen with regard to their ability to produce TNF-α, on the contrary, increased rates of IL-17^+^ MAIT cells in PB of AS samples were demonstrated [88]. Moreover, IFN-γ^+^ and TNF-α^+^ MAIT cells were significantly decreased in AS SF, in contrast with enhanced IL-17 and granzyme production [87]. It is also worth noting that increased circulating MAIT cells producing IL-22 were found in PB of axial-SpA patients [89]. Moreover, MAIT cells in AS show elevated IL-17 production following stimulation with IL-7, while it seems to be not dependent on IL-23 action [87]. Rosine et al. showed that both peripheral blood and resident MAIT cells in enthesis of non-axial-SpA subjects were capable of producing IL-17 even more than T_H_17 cells, thus suggesting their critical role in disease-affected tissue [64].

Invariant Natural Killer T cells (iNKT) are another type of *ILLs.* These are CD1d-restricted T cells expressing a semi-invariant TCR that can recognize glycolipid antigens bound to an MHC class-1 like glycoprotein called CD1d [90]. A small part of iNKT can express RORγT transcription factor and IL-23R, being able to produce IL-17A and IL-22 after TCR and IL-23 stimulation [84]. Moreover, iNKT seem to have regulatory actions on murine models of SpA, as in TNF-α ΔARE/+ mice where prolonged exposure to TNF-α action was able to induce iNKT activation, reducing gut and joint inflammation [91]. iNKT were also seen to have a protective role against *Salmonella*-induced reactive arthritis due to downregulation of IL-17-producing-γδ T cells [92]. On the contrary, an IL-17 producing iNKT population was demonstrated in the joints of SpA [93]. Further studies are warranted to better elucidate the actual protective or pathogenic role of this population in axial-SpA pathogenesis.

Moreover, γδ T cells also seem to be involved in disease pathogenesis, especially the γδ_17_ T cells, a subtype sharing common features with other IL-17-producing cells such as the transcription factors RORγt and STAT3 as well as IL-23R and CCR6 [84,94]. In this regard, Kenna et al. demonstrated the enrichment of IL-23R^+^ IL-17-producing γδ T cells in AS PB, combined with the increased responsiveness of these cells to IL-23 [95]. Their actual presence in human normal enthesis has been recently shown, witnessing their possible involvement in the pathogenic process of enthesitis. It was seen in entheseal tissue that these cells can also produce IL-17 independently of IL-23, an act providing a possible explanation for IL-23 blockers ineffectiveness in axial-SpA [96]. In an IL-23 overexpression murine model similar to human AS-associated inflammation, an IL-23R^+^ RORγt^+^ CD3^+^ CD4^-^ CD8^-^ population, mainly composed of γδ T cells and able to produce IL-17 and IL-22, was found to induce entheseal inflammation and bone remodelling [97]. Nevertheless, in mice overexpressing IL-23, γδ T cells can accumulate at the Achilles tendon enthesis, but also in other typical axial-SpA-targeted tissues such as within the uvea, especially near the ciliary body and within the aortic valve and root [98]. Further proof supporting the importance of the gut–joint axis in the SpA pathogenetic process is the evidence that in the PB of patients compared with controls, even when the total number of γδ T cells was reduced, there was an increased frequency of γδ T cells expressing on their surface the intestinal homing receptor α4β7. In this regard, these cells, once activated in the gut due to a microbiome impairment, may migrate to peripheral tissues such as joints, where high levels of MAdCAM1 could regulate this process [99]. The role of the innate immune system in axial-SpA pathogenesis is depicted in Figure 2.

## 4. Role of Adaptive Immunity

The role of T cells in axial-SpA has been largely evaluated, albeit many questions still remain unsolved. As previously mentioned, the strong correlation between HLA-B27 and disease onset suggests a putative cardinal role of CD8^+^ T cells. Nevertheless, in HLA-B27/human-β2-microglobulin-transgenic rats, it has been found that disease manifestations develop in the absence of any functional CD8^+^ T cells, thus questioning their importance in the pathogenetic process [100]. Conversely, in another murine model, the transfer of CD4^+^ T cells from SKG mice to lymphopenic Severe Combined Immunodeficiency (SCID) recipients caused the development of arthritis and spondylitis [101]. This evidence enlightens the role of T cells and autoimmunity as a sufficient component involved in SpA pathogenesis. There is evidence about the role of αβ T cells derived from Zheng et al., who found that a predominant rate of CDR3 amino acid sequences and full TCR sequences could be identified in CD8^+^ T cells as well as in CD4^+^ T cells at the same time in axial-SpA patients with highly active disease. These cells with different CD surface molecules but the same TCR sequences were named “CD4/8” T cells. A significant percentage of expanded clonotypes depended on CD4/8 T cells, where these clonotypes share common CDR3 sequences, probably due to antigenic stimulation. Moreover, they found identical CD4/8 T cells among different patients, and in every patient these cells were well-represented. This interindividual sharing of CD4/8 T cell receptors points to the importance of the arthritogenic peptide theory, underlining the likely role of shared antigenic stimulation between CD4^+^ and CD8^+^ populations. This also emphasizes the possibility of HLA-B27 recognition by CD4^+^ T cells with equal TCRs. Suggestive hypotheses about the origin of these CD4/8 T cells have been made. It would seem that thymic selection is critical in the genesis of HLA-B27-recognizing CD4^+^ T cells. The exact mechanism determining thymic differentiation versus CD4^+^ or CD8^+^ cells is not well-clarified. It is true that when the CD4^+^ CD8^+^ double positive thymocytes downregulate CD8 expression, a long-lasting TCR stimulation with positive selecting ligands [102] or a deficient function of the transcription factor MAZR [103] could lead to the selection of HLA-B27 restricted CD4^+^ T cells. Nevertheless, CD4/8 T cells have been found to be strictly correlated to disease activity, thus suggesting adaptive immunity could be pivotal in AS disease progression [104]. Another recent study has demonstrated that the SF of SpA patients hosts CD4^+^ T cells and CD8^+^ T cells with low clonal heterogeneity and large clonal expansions, distinct from those found in PB. Moreover, the same clonotypes may be found in the synovial fluid months after the first evaluation, underlining that they can persist in this tissue as resident T cells. Several TCRβ motifs have been identified to be associated with SpA risk HLA-I alleles, suggesting HLA-I-driven antigenic selection of T cells, also reproducible in SF samples from different patients [105]. In addition, CD8^+^ TCR of AS patients also showed a possible involvement of viral antigens, based on a revealed expansion of Epstein–Barr virus and Cytomegalovirus specific clonotypes [26]. Another corroboration of the cytotoxic T cells role relies on the presence of CD8^+^ T cells with an activated cytotoxic profile more prominent in the joints than in the PB [22].

Several studies were performed on T/B cell proportions in AS patients, proving a disequilibrium between distinct subpopulations, especially in the PB [106,107,108,109]. First of all, a disequilibrium between T_H_17 and T_REG_ is now commonly accepted as an important axial-SpA feature. Indeed, T_H_17 levels are increased in AS PB coherently with the critical role of this lineage and IL-17-producing cells in AS pathogenesis. Moreover, some sex-related differences have been found about this population: in particular, T_H_17 cells seem to be increased in male AS patients, hence, setting the preconditions for sex-specific therapeutic consequences [108,110]. In contrast with the radiographic form, circulating T_H_17 cells are significantly reduced in patients with early nr-axial-SpA [111], raising the question of whether they are involved only in advanced phases of the radiographic progression of the disease. Additionally, T_H_17 cells are essential for the mucosal defence against extracellular bacteria and fungi through the stimulation of a significant neutrophilic inflammatory response and the production of several antimicrobial substances from various cells. Their differentiation is promoted by bacteria and fungi-induced cytokines such as IL-6, IL-1, and TGF-β, in turn secreted by dendritic cells in response to these microbes. These cytokines are capable of inducing RORγT and STAT3 transcription factors essential for T_H_17 cell development and the production of signature cytokines such as IL-17A, IL-17F, and IL-22 [112]. Accumulation of T_H_17 in AS seems to be elicited also by arachidonic acid derivatives, such as prostaglandin E_2_ (PGE_2_). In this regard, arachidonic acid derivatives play an important role in disease-mediated inflammation, as confirmed by the inhibition of cyclooxygenase through nonsteroidal anti-inflammatory drugs (NSAIDs) [113]. Hence, PGE_2_ can act on these cells through surface receptors such as EP_2_ and EP_4_. GWAS studies identified some AS risk alleles in the EP_4_ gene *PTGER4*; on this basis, Klasen et al. evaluated its possible role in T_H_17 AS accumulation. PGE_2_ may induce T_H_17 development, acting through EP_4_ receptor, which in turn signals via cAMP and the PI3K/Akt pathway in parallel. The PI3K/Akt pathway regulates T_H_17 cell differentiation thanks to the inhibition of FoxO1; the latter leads to the upregulation of IL-23R, increasing these cells proliferation [114].

Among T cells, not only is T_H_17 pivotal in the host mucosal defence, but also tissue-resident memory T cells T_RM_ are an important component of this first adaptive defensive line in the human body. The strong link between gut and joint is also reinforced by the expansion of T_RM_ cells in the PB, gut, and SF of AS patients. These cells express the α4β7 integrin, suggesting, even in this case, a gut provenance and a possible migration from the gut to joints [115,116]. Even if IL-17-producing cells also constitute the main AS pathogenetic actor, other T cell subtypes seem to contribute. In particular, T_H_22 cells with their isolated IL-22 production [108] and T_H_1 cells have been found to be increased, especially in subjects with active disease [107,117].

The disequilibrium between effector components of adaptive immunity and their regulatory counterpart, T_REG_ cells, is one of the most important features of AS pathogenesis. Thus, AS patients have quantitative and qualitative T_REG_ alterations when compared with healthy controls. Regarding the quantitative aspects, several recent studies suggest a global reduction of active T_REG_ cells in AS patients [106,107,109,118]. Moreover, there is also a significant qualitative reduction in the efficacy of their anti-inflammatory role in axial-SpA patients. T_REG_ cells in AS appear to be less sensitive to IL-2 as well as to have decreased STAT5 phosphorylation and epigenetic inhibitory changes such as higher CpG methylation levels in the *foxp3* gene CNS2 region [119].

In conclusion, adaptive T immunity is clearly dysregulated toward a proinflammatory phenotype, demonstrating its key role, particularly in disease progression. Moreover, new regulators of T cells and, thus, possible therapeutic targets, have been emerging in the last few years. Among them, semaphorine 4D seems to be involved not only in rheumatoid arthritis (RA) pathogenesis but also in AS immune dysregulation, being able to act through the aryl-hydrocarbon receptor AhR pathway, thus increasing T_H_17 response and inhibiting the T_REG_ counterpart [120].

B lymphocyte involvement in axial-SpA has traditionally been considered minimal, although a possible role for humoral immunity marked by B cells infiltration in disease-affected tissues has been suggested [121]. Some evidence shows that B cell subtypes distribution in AS patients is impaired when compared to healthy subjects as well as their percentages being increased in patients with active disease [106,122]. Contrasting data about B_REG_ cells suggest that they are not reduced [123], while their functionality may be impaired due to reduced IL-10 production [124]. Furthermore, activated B cells with upregulated expression of CD86 or CD95 seem increased in AS patients, along with T_FH_ cells, which are crucial in humoral immunity activation [125]. Despite these assumptions, B cell-targeted therapy with anti-CD20 antibodies has proven to be ineffective in these patients [126,127], with the exception of a subset of patients naïve to TNF-α inhibitors [128]. The lack of efficacy of this targeted therapy underlines how B cells involvement in axial-SpA could be limited.

Although traditionally classified as seronegative arthritis, several autoantibodies have been demonstrated in serum from axial-SpA patients [125]. The best-characterized autoantibody in these patients is the anti-CD74, able to bind the MHC class II histocompatibility antigen invariant gamma chain CD74. In plain words, CD74 prevents the premature binding of peptides to the peptide-binding groove in MHC class II molecules thanks to a small fragment of the extracellular domain of the molecule, known as class II-associated invariant chain peptide (CLIP). CD74 also represents the surface cellular receptor for the cytokine MIF [129]. Formation of these autoantibodies could be linked to the reduced activity of the enzyme signal peptide peptidase-like 2A (SPPL2A), which in turn may cause the accumulation of CD74 and its degradation products, thus inducing antibody production [130]. The role of these autoantibodies in axial-SpA is currently unknown, but those directed towards the CLIP part of the molecule could be used as serum biomarkers to disclose the disease in these patients. Indeed, anti-CLIP antibodies would appear to have good specificity. In a group of 145 patients (94 axial-SpA and 51 not-SpA patients), these antibodies were found only in 7,8% of not-SpA patients while being detectable in 85,1% of axial-Spa serum with a specificity of 92.2% [131].

Autoantibodies against a peptide of a *Klebsiella pneumoniae*-derived protein have also been found in the serum of AS patients. In fact, serum IgG antibodies against *Klebsiella pneumoniae* dipeptidase protein (DPP) were demonstrated to be in 95% of AS patients, in contrast with 1.5% of AR patients and 1% of psoriatic arthritis (PsA) patients. The most interesting finding about these antibodies is that DPP protein from *Klebsiella pneumoniae* has some similarities with various molecules highly concentrated in fibrocartilaginous tissues such as collagen type I, collagen type II, and fibronectin, which may be recognized by these cross-reacting antibodies [132]. Antisclerostin and antinoggin autoantibodies represent other particular antibodies detected in serum from AS, albeit their pathogenic role is not determined yet [133]. Sclerostin and noggin are two important regulators of bone homeostasis: both may inhibit bone formation through different modalities [134,135]. Low serum sclerostin has been seen in axial-SpA patients [136]; perhaps it is conceivable that immune complexes of antibodies and these two proteins could reduce sclerostin and noggin serum levels, thus enhancing dysregulated new bone formation. However, further studies are surely needed to better elucidate the role of these antibodies. The role of the adaptive immune system in axial-SpA pathogenesis is depicted in Figure 2.

## 5. Therapeutic Targets in Axial-SpA

### 5.1. TNF-α

**Figure 3:**
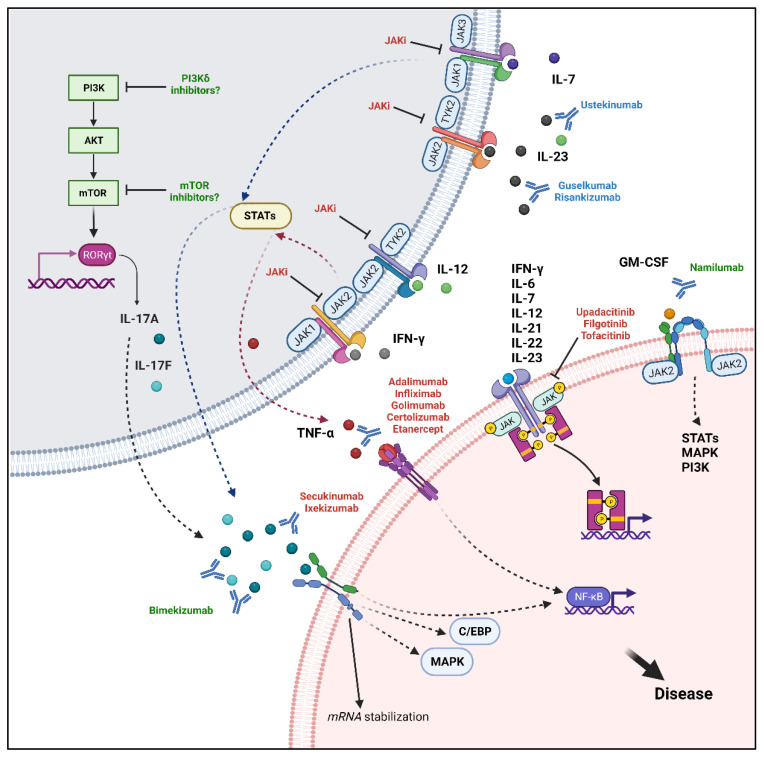
Molecular targets and associated signalling pathways. Current therapeutic targets in axial-SpA include TNF-α, IL-17A, and JAKs. TNF-α signals mainly through the NF-κB pathway and could be inhibited by several monoclonal antibodies, anti-TNF-α, and the fusion protein etanercept. IL-17A has a pivotal role in mediating disease pathogenesis and could activate several pathways such as the canonical NF-κB, MAPK, C/EBP pathways and the alternative stabilization of mRNA. Secukinumab and ixekizumab can bind IL-17A with different affinities, inhibiting its downstream actions. The JAK inhibitors upadacitinib, filgotinib, and tofacitinib may act directly by blocking the signal transduction of proinflammatory cytokines including IFN-γ, IL-6, IL-7, IL-12, IL-21, IL-22, IL-23, or indirectly blocking TNF-α and IL-17A/F production following inhibition of upstream stimuli. New targets include the simultaneous inhibition of IL-17A and IL-17F with bimekizumab. Moreover, a potential therapeutic strategy may be represented by GM-CSF inhibition through namilumab. Finally, the PI3K/Akt/mTOR pathway has important immunomodulatory roles, it can elicit RORγT translocation into the nucleus, promoting IL-17A/F production and could be further investigated as a novel target. “Created with BioRender.com”.

TNF-α represents a key cytokine involved in the inflammatory process of axial-SpA. Its production is related to various immune cells, in particular activated macrophages and T-lymphocytes. TNF-α exists in two different forms: a transmembrane form and a soluble form. TNF-α is initially synthesized as a transmembrane protein (tmTNF-α), which, following proteolytic cleavage by the TNF-α-converting-enzyme (TACE), is released as the soluble TNF-α (sTNF-α). This cytokine may then signal through two receptors (TNFR1 and TNFR2) and lead to the activation of the Nf-κB or to apoptosis, depending on the metabolic state of the cell [137]. Under physiologic conditions, TNF-α is a key factor for the proper functioning of the human immune response. However, aberrant TNF-α production could lead to tissue damage because of an abnormal immune response. For this reason, TNF-α inhibitors have been largely used throughout recent history for managing inflammatory diseases such as SpA, RA, psoriasis, inflammatory bowel disease IBD, and juvenile idiopathic arthritis [138]. These therapeutic agents include monoclonal antibodies such as infliximab, adalimumab, golimumab and certolizumab pegol, and a fusion protein, etanercept. The key role of TNF-α in axial-SpA was first demonstrated directly in sacroiliac joint biopsy specimens from AS patients [139]. Furthermore, *TNF-α* mRNA was found to be significantly overexpressed in PBMCs of AS patients compared to HD, positively correlating its levels with disease activity [140]. More recently, Christodoulou-Vafeiadou et al. reproduced axial-SpA features, including new bone formation and ankylosis, using a tmTNF transgenic mouse model that further confirmed the pathogenic role of this cytokine in axial-SpA [141]. On this basis, the benefit of TNF inhibitors has been widely demonstrated in several clinical trials [142,143,144,145,146,147,148]. Moreover, recent studies have also highlighted the usefulness of TNF inhibitors on radiographic progression in long-term follow-up [149,150,151]. Figure 3 shows the main molecular targets in axial-SpA.

### 5.2. IL-17

IL-17 represents a family of cytokines including IL-17A, IL-17B, IL-17C, IL-17D, IL-17E, IL17F, with IL-17A being the best characterized and studied. IL-17F shares 50% homology with IL-17A, and these two related cytokines may exist as IL-17A homodimers, IL17-F homodimers, and IL-17A/F heterodimers [152]. Traditionally considered as the main CD4^+^ T_H_17 product, IL-17A may be actually produced and secreted by a heterogenous cellular cluster, also including CD8^+^ cells, γδ T cells, iNKT cells, MAIT cells, *ILC3* cells, and T_RM_ cells; all IL-17 producing cells depend on the activity of the transcription factor RORγT [82,93,96,153,154,155]. Data about IL-17 neutrophil production are, on the other hand, contrasting at the moment [64,66]. IL-17A/F homodimers and heterodimers signals through a heterodimeric receptor known as IL-17RA/RC. IL-17A can induce a stronger activation of the downstream pathway than IL-17F, thus explaining its most relevant role in axial-SpA pathogenesis. Receptor binding elicits the activation of various canonical pathways such as the NF-κB, mitogen-activated protein kinase (MAPK), and CCAAT-enhancer-binding proteins (C/EBPs) pathways. Hence, IL-17A upregulates de novo gene transcription of inflammatory genes. Moreover, an alternative pathway that may lead to the stabilization of proinflammatory cytokines and chemokines mRNA is elicited [152]. Therefore, IL-17A action results in the production and secretion of various proinflammatory cytokines, including TNF-α, IL-6 and IL-1, proinflammatory chemokines, antimicrobial peptides, and growth factors such as granulocyte colony-stimulating factor (G-CSF) and granulocyte macrophage-colony stimulating factor (GM-CSF). Moreover, aberrant secretion of IL-17A in inflammatory arthritis may lead to the production of matrix metalloproteinases (MMPs) and the promotion of angiogenesis, driving inflammatory cells to the affected tissue [152,156]. Traditionally, IL-23 was thought to be one of the crucial cytokines for IL-17 production, with the IL-23/IL-17 axis being considered pivotal in axial-SpA immunopathogenesis. IL-23 is a heterodimeric cytokine made up of a p40 chain, common to the heterodimeric structure of IL-12, and a p19 chain that alone has no determined biological activity. This cytokine is produced by antigen-presenting cells, largely dendritic cells, monocytes, and macrophages, and it binds to its receptor complex composed of two subunits, IL-12Rβ1 and IL-23R, respectively associated with Janus Kinases TYK2 and JAK2. JAKs activation leads to the phosphorylation of signal transducer and activator of transcription (STAT) proteins, mainly STAT3. Activated pSTAT3 translocates into the nucleus and induces the transcription of several inflammatory cytokines, such as IL-17A and IL-17F [157,158,159]. Genetic studies showed several associations between the IL-23/IL-17 pathway and axial-SpA. In this regard, association with axial-SpA was found in variants of the *IL-12 p40* subunit, *IL-23 p19* subunit, and *IL-23R* locus. Furthermore, AS susceptibility variants were recognized in IL-23 downstream molecules, including *TYK2* and *STAT3,* as well as in *IL-17A*, *IL-17F* and *IL-17RA* [160,161,162,163]. Interestingly, HLA-B27 seems to contribute to the upregulation of the IL-23/IL-17 pathway through two distinct mechanisms: (1) UPR and (2) KIR3DL2 binding of HLA-B27 homodimers. Moreover, a recent meta-analysis has shown that IL-17 levels are increased in AS from the PB when compared to the HD. Of note, these levels are significantly higher in patients with active disease compared to those with inactive AS [164]. In addition, a higher number of IL-17-secreting cells have been found in AS facet joints than in those of subjects with osteoarthritis [65].

The role of IL-17A in axial-SpA is quite pleiotropic. This cytokine affects bone homeostasis in the AS microenvironment in a contrasting way, modulating bone resorption and new bone formation. Indeed, AS is associated with bone loss in both the trabecular and cortical bone [157,165], and IL-17A may drive this process by stimulating the receptor activator of nuclear factor-κB ligand (RANKL) expression on osteoblasts, thus promoting osteoclast differentiation and bone resorption [166,167]. Beyond the IL-17A-mediated osteoclastogenesis process, this cytokine may also affect new bone apposition. In fact, axial-SpA is characterized by new bone deposition at the entheseal site. This bone tissue comes from an endochondral bone formation process following previous disease-induced damages in these anatomical sites. IL-17A, as well as NET-associated IL-17A, seems to increase osteoblastogenesis through osteoblast differentiation of MSCs in affected anatomical tissues, albeit it also has inhibitory effects on already committed osteoblasts [66,168,169,170,171,172]. Jo et al. showed that IL-17-dependent osteoblastogenesis is marked by upstream activation of the JAK2/STAT3 pathway, as JAK2 inhibition was able to downregulate the increase in alkaline phosphatase (ALP) activity mediated by AS patient serum [173]. Moreover, Daoussis et al. found that IL-17A significantly downregulated the expression of Dkk-1 genes, which could inhibit, in turn, the Wnt pathway in both MSCs and osteoblasts from AS patients [174]. The activity of IL-17A on bone homeostasis could be reinforced by TNF-α through a synergic effect with the former [175]. Bone destruction may be exacerbated in the presence of both IL-17A and TNF-α [167], but, paradoxically, these two cytokines may also synergize in promoting new bone formation in AS. Whether the final effect of their synergism results in bone destruction or new bone formation may probably depend on the microenvironment in which cellular–cytokine interactions take place [176]. Overall, from a molecular point of view, this synergism with TNF-α seems to depend upon the mRNA transcript stabilization induced by IL-17A, in turn regulated by p38 MAPK activation [177].

IL-17 also has a key role in pain modulation in axial-SpA. Indeed, several studies showed that axial-SpA patients experience phenomena of central sensitization and neuropathic pain that may significantly worsen their quality of life [178,179,180,181,182,183]. A recent meta-analysis showed that in a group of 1483 AS patients, 41,5% of them had likely neuropathic pain symptoms, thus, showing the relevance of the phenomenon [184]. Several data prove that inflammatory pain, central sensitization, and neuropathic pain may all be modulated, at least in part, by IL-17A. In this regard, IL-17A may act directly on the neuronal pathways or indirectly induce hyperalgesia depending on the amplification of several proinflammatory factors [185,186]. IL-17R has been found in Dorsal Root Ganglia (DRG) neurons of experimental models and IL-17A has the potential to act on nociceptive neurons, inducing inflammation-dependent mechanical hyperalgesia [187]. Several mechanisms seem to contribute to IL-17 dependent hyperalgesia. In fact, IL-17 is able to increase tetrodotoxin (TTX)-resistant sodium currents in murine models, whereas IL-17A KO eliminates mechanical hyperalgesia in Antigen-induced Arthritis (AIA), showing the strong connection between mechanical hyperalgesia and IL-17A [188]. Another important effect of IL-17A is the selective Transient Receptor Potential Cation Channel Subfamily V Member 4 (TRPV4) upregulation in DRG neurons, this receptor being able to transmit noxious mechanical stimuli [189]. Moreover, IL-17A may upregulate NR1 subunit phosphorylation, hence enhancing N-methyl-D-aspartate (NMDA) receptor activity and changing neuron sensitivity [190].

Due to its pleiotropic involvement in axial-SpA pathogenesis, IL-17A blockade has become a therapeutic landmark over the last few years [191,192,193,194,195,196,197,198]. Approved IL-17A inhibitors include secukinumab and ixekinumab; both are monoclonal antibodies able to bind this cytokine and block its activity, with the latter having a greater affinity for IL-17A [199]. They showed efficacy versus placebo in both naïve patients and those with inadequate response to TNF inhibitors. Moreover, these therapeutic agents have proven not only to be capable of improving symptoms and disease activity, but also to slow the radiographic progression. In this regard, a 4-year lasting study showed that secukinumab administration led to no radiographic progression in almost 80% of patients [200]. Recent evidence showed, in addition, a possible pathogenetic role also for the homologous cytokine IL-17F. Indeed, IL-17F levels are higher in AS patient serum; this cytokine may stimulate in vitro osteogenic differentiation and bone formation, acting synergistically with IL-17A in new bone formation [201]. On this basis, the monoclonal antibody neutralizing both IL-17A/F, bimekizumab, was shown to be effective in phase II and III clinical trials [202,203,204]. Figure 3 shows the main molecular targets in axial-SpA.

### 5.3. IL-23: A Debated Role in Axial-SpA

Due to the relevance of the IL-23/IL-17 axis in disease pathogenesis and the association between axial-SpA patients and gene variants encoding for the main component of this axis, IL-23 blocking was expected to have clinical efficacy in axial-SpA, similar to IL-17 inhibitors. Unexpectedly, the administration of IL-23 inhibitors, such as risankizumab, ustekinumab, or guselkumab, was not as effective as expected. Thus, clinical trials with these monoclonal antibodies did not demonstrate great improvement in clinical features and MRI inflammation in axial-SpA [205,206]. On the other hand, IL-23 inhibitors showed their efficacy in treating peripheral PsA and peripheral enthesitis, thus raising several questions about why these therapeutic agents would work less well in axial-SpA [207,208,209,210]. One possible explanation for these discrepancies is represented by different anatomical features of peripheral and axial enthesis. Nevertheless, various synovio–entheseal complexes could be found in the peripheral skeleton, rich in myeloid cells, whereas the latter are rare in the axis. Peripheral enthesitis is also characterized by a more important inflammation of entheseal soft tissue, while axial enthesitis implies a major involvement of the perientheseal bone [211]. Although IL-23 myeloid-producing cells have been recently demonstrated in the spine [72], it is possible that IL-17 production in these two anatomical sites could be regulated in different ways, thus making the peripheral involvement more dependent on IL-23. However, blocking IL-23 has shown to be also effective for axial PsA [212,213]. It is conceivable that not only anatomical differences affect the therapeutic response but also a heterogenous immunological process that relies on different cytokines and cellular pathways. In fact, it would seem that axial-SpA is based mainly upon an IL-23-independent IL-17-driven process. Several studies demonstrated how immune cells could produce IL-17 thanks to IL-23 alternative stimuli. Therefore, spinal enthesis hosts two different kinds of γδ *T* cells including γδ1 T cells and γδ2 T cells. The former population does not express IL-23R and is able to produce IL-17A upon phorbol myristate acetate (PMA) or anti-CD3/CD28 stimulation [96]. Moreover, MAIT cells, one of the most relevant IL-17-producing cells in axial-SpA, can secrete IL-17 independently of IL-23. Gracey et al. demonstrated in PBMCs from AS that IL-7 is able to increase IL-17 MAIT production with a higher effect than IL-23. Interestingly, PBMCs from AS were more sensitive to IL-7-driven IL-17 production than HD PBMCs, thus implying that this cytokine could at least partially explain why IL-23 does not have a key role in axial-SpA [87]. Other authors showed that MAIT cell-IL-17A/F production could be induced by prolonged IL-12 and IL-18 stimulation, independent of IL-23. Moreover, *ILC3* cells have also been found to produce IL-17A/F independently of IL-23 [214]. Although IL-23 could be not critical in perpetuating the pathogenetic process once established, it is possible that it plays a key role in disease onset. Evidence on animal models showed that early IL-23 inhibition in Curdlan-induced SpA in SKG mice significantly suppressed the axial disease [215] and an anti-IL-23R prophylactic therapy completely prevented Mycobacterium tuberculosis-induced spondylitis and arthritis in HLA-B27 transgenic rats. Conversely, when the same rats were treated with IL-23R inhibitors following clinical onset, no disease suppression occurred, similar to what happened in the clinical trials of IL-23 blocking agents [216]. Figure 3 shows the main molecular targets in axial-SpA.

### 5.4. JAK–STAT Pathway

Currently, greater importance is given to intracellular pathways mediating downstream actions of multiple ligands. Hence, multiple cytokines, chemokines, growth factors, and other molecules lead to the activation of the JAK–STAT pathway, which is involved in the pathogenetic mechanism of axial-SpA. The Janus Kinases proteins are four distinct tyrosine kinases: JAK1, JAK2, JAK3, and TYK2; each of them can associate with the intracellular domain of class I and II cytokine receptors. When cytokines interact with their receptors on the cell surface, they elicit receptor dimerization, thus leading to the transphosphorylation of JAKs homo- or heterodimers and the associated receptors. Then, STAT proteins interact with this complex; they are phosphorylated by the JAKs and form STAT homo- or heterodimers that can migrate from the cytosol to the nucleus, hence regulating gene expression as a transcription factor [217,218]. Inhibition of JAKs is able to interfere with the signalling of several cytokines at one time, in contrast with what happens when using TNF or IL-17 inhibitors, thus leading to alternative therapeutic opportunities blocking multiple potential targets at the same time [219]. Of note, cytokines could exploit this pathway directly or indirectly. Accordingly, in axial-SpA JAK inhibitors (JAKi) could directly block the action of pivotal cytokines such as IFN-γ, IL-6, IL-7, IL-12, IL-21, IL-22, and IL-23, signalling through the JAK/STAT pathway with different pairs of JAK molecules. On the contrary, TNF-α and IL-17 do not directly stimulate this pathway and could be modulated indirectly by JAKi. In fact, IL-23- and IL-7-dependent IL-17 production may be affected by the inhibition of JAK2/TYK2 and JAK1/JAK3 heterodimers, respectively. At the same time, macrophage TNF-α production is strictly related to IL-12 and IFN-γ; therefore, blockade of JAK2/TYK2 and JAK1/JAK2, respectively, will downregulate TNF-α action [220].

The involvement of this pathway in AS is suggested by genetic studies that have recognized polymorphism in JAK2, TYK2, and STAT3 as susceptibility factors for AS onset [221,222]. Moreover, JAK/STAT pathway involvement has been confirmed by preclinical and clinical evidence. Indeed, blocking this pathway through JAKi in animal models such as the SKG mice is able to suppress both inflammation and periosteal/entheseal bone formation [223]. Additionally, Hammitzsch et al. demonstrated that inhibition of JAKs in CD4^+^ T cells downregulated several T_H_17 cytokines such as IL-17A, IL-17F, and IL-22 [224]. Currently, the JAKi tofacitinib, upadacitinib, and filgotinib have been evaluated in axial-SpA patients showing meaningful results. Each of them has a distinct selectivity towards the various JAKs. Tofacitinib is capable of inhibiting mainly JAK1 and JAK3, with less selectivity for JAK2, whereas upadacitinib and filgotinib are JAK1 selective inhibitors [225]. JAKi efficacy in AS was confirmed by phase II and III trials [226,227,228,229]; of note, upadacitinib has shown to be effective also in the treatment of nr-axial-SpA [230]. Figure 3 shows the main molecular targets in axial-SpA.

### 5.5. Future Perspectives: Potential Therapeutic Targets

Despite the progress in axial-SpA management, a non-negligible percentage of patients do not respond or become intolerant to currently available treatments. Therefore, there is an actual need for novel therapies: several pathogenic factors could be theoretically investigated as potential therapeutic targets. Among these, the granulocyte-macrophage colony-stimulating factor (GM-CSF), a pleiotropic proinflammatory cytokine and haematopoietic growth factor that seems to contribute to the tissue damage in axial-SpA, could be taken into account. This cytokine exerts its function through the activation of the GM-CSF receptor, thus activating the JAK/STAT pathway via JAK2 homodimers, the MAPK and PI3K pathway [231]. Therefore, GM-CSF proinflammatory function could be inhibited by JAKi acting on JAK2. GM-CSF serum levels have been found to be increased in patients with active disease and correlate with disease activity [232]. Moreover, GM-CSF-producing T cells have been seen to be expanded in SpA joints. In particular, GM-CSF acts on myeloid cells such as monocytes, macrophages, and dendritic cells promoting their differentiation towards a more proinflammatory phenotype. It may promote joint damage by recruiting inflammatory cells from the adjacent bone marrow and may increase bone resorption through the induction of matrix metalloproteinases and the pro-osteoclastogenic factor RANKL. Moreover, GM-CSF may also promote the release of proinflammatory chemokines such as CCL17 in an IRF4-dependent pathway. Of note, GM-CSF also seems to contribute to joint pain by enhancing the response of sensory nerve fibres to mechanical stimuli [233]. On this basis, GM-CSF inhibitors may represent a new therapeutic target for managing patients with axial-SpA. In this regard, the monoclonal antibody targeting GM-CSF, namilumab, did not show a significant efficacy over placebo in the phase IIa trial NAMASTE (NCT03622658). Nevertheless, further investigations on GM-CSF inhibitors efficacy against axial-SpA are needed before completely discarding this class of potential new drugs.

Due to the importance of T_H_17 cell response in axial-SpA pathogenesis, other putative targets might be investigated. In this regard, the PI3K/Akt/mTOR pathway may represent a successful therapeutic strategy since the activation of this pathway contributes to the activation of T cells promoting RORγT nuclear translocation which, in turn, is pivotal for IL-17A expression [234]. In addition, this pathway is involved in bone anabolism and could enhance osteoblastogenesis and mineralization [235]. Chen et al. demonstrated that the mTOR pathway is activated in inflamed synovial tissues from SpA patients. Moreover, the use of the mTOR inhibitor rapamycin is able to reduce in vitro production of TNF-α and IL-17A from SpA PBMCs and to downregulate osteogenic differentiation of fibroblast-like synoviocytes (FLS). More importantly, in vivo administration of rapamycin on HLA-B27 transgenic rats has been shown to be effective both on the inflammatory joint disease and bone alterations [236]. mTOR activation depends on the action of other upstream enzymes, especially the phosphoinositide3-kinase (PI3K). Interestingly, blocking in vitro the PI3Kδ with the selective PI3Kδ inhibitor seletalisib, in HD and SpA samples, has proven to reduce the production of several proinflammatory cytokines, including IL-17A and IL-17F, from both adaptive (T_H_17 cells) and innate-like lymphocytes (MAIT cells and γδ T cells) [237]. However, studies on murine models are lacking and thereby needed, since HLA-B27 transgenic murine models showed an unexpected inefficacy of RORγT inhibition, which enhanced the occurrence of experimentally induced arthritis in contrast with in vitro data [238]. New and potential therapeutic targets of axial-SpA are showed in Figure 2, whereas their mechanism of action and clinal stage are summarized in Table 1.

## 6. Closing Remarks

Currently, it is well-known how deeply complex the etiopathogenic process of axial-SpA is. The disease is the result of tangled interactions deriving from genetic features, immunological cells and molecules, biomechanical joint stress, and other environmental factors, among which the gut microbiome seems to play a critical role [239,240]. Microbial molecules and metabolic products may thereby be key regulators of several immune cells, ready to recognize them. Among these immune cells, *ILCs* and *ILLs,* as well as T_RM_ lymphocytes may play an important role alongside more traditional innate and adaptive cells. Hence, the outcome of these complex cellular and molecular interactions is inflammatory damage and in-depth bone remodelling, which could be targeted through TNF, IL-17A/F, and JAKs blocking agents. Since nonresponders or intolerant patients are not rare, there is a need for new therapeutic solutions. Therefore, new targets such as GM-CSF and the PI3K/Akt/mTOR pathway could be evaluated as potential therapeutic strategies. Axial-SpA is often partnered by extra-articular manifestations such as intestinal and uveal inflammation representing a further disease burden that may require a tailored treatment. In conclusion, although much progress has been made in the comprehension of pathogenic mechanisms of axial-SpA, we are still far from fully understanding the intricate cytokine networks and cellular pathways leading to the development of the disease. Looking into the future, our goal must be to unravel the complex and tangled underworld of axial-SpA, translating our knowledge on disease pathogenesis into a patient-tailored treatment.

## Figures and Tables

**Table 1:** Mechanism of action and clinal stage of new and potential therapeutic targets in axial-SpA.

Target	Drug/Inhibitor	Clinical Stage
TNF-α	Infliximab, adalimumab, golimumab, certolizumab etanercept	Efficacy confirmed in phase III clinical trials [142,143,144,145,146,147,148]
IL-17A IL-17A/F	Secukinumab, ixekizumab Bimekizumab	Efficacy confirmed in phase III clinical trials [191,192,193,194,195,196,197,198] Efficacy confirmed in phase III clinical trials [204]
IL-23	IL-23/12 p40 inhibitor: ustekinumab IL-23 p19 inhibitor: risankizumab, guselkumab	Efficacy in axial-SpA was not confirmed in phase III clinical trials [205] Post hoc analysis from phase III clinical trials showed efficacy in axial-PsA [212] Efficacy in axial-SpA was not confirmed in a phase II clinical trial [206] Post hoc analysis from phase III clinical trials showed efficacy in axial-PsA [213]
JAK/STAT pathway	JAKs inhibitors: tofacitinib, upadacitinib, filgotinib	Efficacy confirmed in phase III clinical trials [226,227,228,229,230]
GM-CSF	Namilumab	Efficacy was not confirmed in a phase IIa clinical trial (NCT03622658)
PI3K/Akt/mTor pathway	PI3Kδ inhibitor (seletalisib) mTor inhibitor (rapamycin)	NA NA

## Data Availability

Not applicable.
